# *Helicobacter pylori* Seroprevalence and Its Association with Gastrointestinal Symptoms and Self-Perceived Oral Health Among Lithuanian Dental Students

**DOI:** 10.3390/diagnostics16071049

**Published:** 2026-03-31

**Authors:** Eglė Slabšinskienė, Rūta Grigalauskienė, Marija Kurenkovienė, Nikolajus Kurenkovas, Laimas Virginijus Jonaitis, Ingrida Vasiliauskienė, Aistė Kavaliauskienė

**Affiliations:** 1 Department of Preventive and Paediatric Dentistry, Faculty of Odontology, Medical Academy, Lithuanian University of Health Sciences, J. Lukšos-Daumanto St. 6, LT-50106 Kaunas, Lithuania; ruta.grigalauskiene@lsmu.lt (R.G.); ingrida.vasiliauskiene@lsmu.lt (I.V.); 2 Faculty of Medicine, Medical Academy, Lithuanian University of Health Sciences, Mickevičiaus St. 9, LT-44307 Kaunas, Lithuania; 3 Department of Gastroenterology, Faculty of Medicine, Medical Academy, Lithuanian University of Health Sciences, Mickevičiaus St. 9, LT-44307 Kaunas, Lithuania; laimas.jonaitis@lsmu.lt; 4 Department of Orthodontics, Faculty of Odontology, Medical Academy, Lithuanian University of Health Sciences, J. Lukšos-Daumanto St. 6, LT-50106 Kaunas, Lithuania; aiste.kavaliauskiene@lsmu.lt

**Keywords:** *Helicobacter pylori*, seroprevalence, dental students, gastrointestinal symptoms, Lithuania

## Abstract

**Background/Objectives**: *Helicobacter pylori* (*H. pylori*) infection remains common globally, yet data on its prevalence and correlates among dental students in Eastern Europe are limited. Dental students may face potential occupational exposure through contact with saliva and aerosols during their clinical training. This study aimed to measure the seroprevalence of *H. pylori* among Lithuanian dental students and evaluate its associations with academic year, self-perceived oral health and hygiene factors, and gastrointestinal symptoms. **Methods**: An observational–analytical cross-sectional study was conducted in 2025 among 202 dental students from lower (I–II) and higher (IV–V) academic years at the Lithuanian University of Health Sciences. Participants underwent serological testing for *H. pylori* IgG antibodies using capillary blood and completed a structured questionnaire on sociodemographic factors, oral health behaviors, clinical exposure, and gastrointestinal symptoms assessed by the Gastrointestinal Symptoms Rating Scale (GSRS). Descriptive and bivariate statistical analyses were performed to assess associations. **Results**: Overall *H. pylori* seroprevalence was 12.4% and did not differ significantly in different academic years. Seropositivity was significantly associated with longer toothbrushing duration and a family history of stomach ulcer. No significant associations were found with the number of patients treated, the use of personal protective equipment, or most oral hygiene indicators. Higher-year students reported greater overall gastrointestinal symptom scores than lower-year students; however, GSRS scores did not differ between *H. pylori*-seropositive and -seronegative participants. **Conclusions**: *H. pylori* seroprevalence in this student population was relatively low, and no association was found with clinical exposure or gastrointestinal symptom severity. Household-related factors may be more relevant to transmission than occupational exposure in dental training. Further longitudinal studies are needed to clarify risk factors and transmission pathways.

## 1. Introduction

The global prevalence of *Helicobacter pylori* (*H. pylori*) is still high, about 44% among adults [1]. It is generally accepted that the acquisition of *H*. *pylori* occurs in early childhood, but some studies showed that seroprevalence increases with age, and the possible acquisition of *H. pylori* in adulthood reinforces the general clinical focus on the adult population and the search for possible new risk factors [2].

Even now, there is no consensus in the scientific community regarding *H. pylori* infection in the oral cavity and gastrointestinal tract. Still, there is a growing body of evidence that suggests that the oral cavity could serve as a *H. pylori* reservoir [3]. Saliva, dental plaque, tongue, root canals, oral mucosa, and tonsillar tissues are recognized as potential sites of *H. pylori* colonization in the oral cavity [4]. The persistence of *H. pylori* in the oral cavity has raised questions regarding the role of oral–gastrointestinal tract health, as it potentially alters oral and gastric microbiomes and could cause significant changes that have broader implications; this suggests that clinical practices could affect pathogen transmission and reinfection, leading to reinfection even after eradication therapy. The data on these issues is controversial because studies investigating *H. pylori* prevalence in the oral cavity and stomach vary depending on their design, diagnostic methods, and populations, which makes comparison of these studies challenging. It has been reported that the prevalence of *H. pylori* infection in the oral cavity ranges from 51.25% to 60.29% [5,6]. There is very limited data on the specific prevalence of oral *H. pylori* infection in Eastern Europe and the Lithuanian population. The most recent epidemiological study conducted among Lithuanian adults indicates that *H. pylori* seroprevalence remains high, with the overall age-standardized *H. pylori* seroprevalence being 63.1% [7].

A seropositive *H. pylori* test does not reliably distinguish between gastric infection and the *H. pylori* presence in the oral cavity. Antibody tests measure response to *H. pylori*, but they do not indicate where in the body this bacterium is or whether the infection is active; thus, they are unable to distinguish between past and active infection [8].

Dental professionals are at an increased risk of exposure to *H. pylori* infection due to their working environment, which involves close contact with patients, aerosols, and saliva [9]. Therefore, we hypothesize that the oral cavity serves as an additional storage site for *H. pylori*. Although transmission of this infection is believed to occur primarily via the oral–oral, fecal–oral, and gastro-oral routes, occupational exposure during dental clinical procedures could present an additional risk factor. Infection risk may be influenced not only by cumulative patient contact but also by adherence to infection control measures.

Dental students represent a unique population in this context. As they progress through their studies, they accumulate clinical experience, modify lifestyle and hygiene habits, and increase exposure to potentially infectious oral environments. Oral health and hygiene habits can significantly influence the transmission of *H. pylori* [10].

Despite growing interest in oral transmission pathways, limited data exist on the prevalence of *H. pylori* among dental student populations or how infection correlates with gastrointestinal symptoms, clinical dental practices, and professional training. Understanding this relationship may help identify risk factors relevant to future dental practitioners and guide preventive strategies within educational and clinical settings.

Building on this rationale, the present study aimed to determine the seroprevalence of *Helicobacter pylori* infection among Lithuanian dental students and explore its associations with clinical exposure during dental training, oral health and hygiene characteristics, and dyspeptic symptoms. The study addressed the following research questions:

(1) What is the seroprevalence of *H. pylori* infection among Lithuanian dental students?
(2) Is seropositivity associated with the level of clinical exposure during dental training?
(3) Is seropositivity associated with family history, oral health, and oral hygiene characteristics?
(4) Is seropositivity associated with dyspeptic symptoms?

## 2. Materials and Methods

### 2.1. Study Ethics

This study was performed at the Lithuanian University of Health Sciences from 1 May to 9 September 2025. It was approved by the Kaunas Regional Biomedical Research Ethics Committee (as from 25 March 2025; approval number BE-2-39). All participants provided written consent, and the study was performed according to the principles of good clinical practice under the Declaration of Helsinki.

### 2.2. Study Design, Population, and Sample Size

An observational–analytical cross-sectional study was conducted among Lithuanian dentistry students from years I, II (lower), IV, and V (higher) of the Odontology Faculty of Lithuanian University of Health Sciences. Third-year dentistry students were not included. All students attending classes on the day of the study were invited to participate. Participation in this study was entirely voluntary. All participants were informed about the purpose, inclusion and exclusion criteria, procedures, and potential risks of the study before providing their written informed consent. They were also assured that they could withdraw from the study at any time without any consequences. Students were eligible for inclusion if they were actively attending the dental program during the study period. Exclusion criteria included antibiotic use within the previous three months, failure to provide written informed consent, a diagnosis of hemophilia, or the presence of any bleeding or coagulation disorders.

The sample size calculation and statistical power analysis were performed using the software G*Power (version 3.1.9.4, University of Dusseldorf, Dusseldorf, Germany) [11]. According to classical sample size estimation, approximately 320 students would be required in each group to reliably detect (α = 0.05; β = 0.20) a difference between 20% and 30% in the prevalence of the factor in two student groups, corresponding to the expected prevalence of a positive *H. pylori* test [12]. However, the total number of students enrolled in the selected academic grades at the university was 295. Therefore, 100 students from the lower and 100 from the higher academic years were included. The post hoc power analysis indicated that, under these conditions, the statistical power of the test was approximately 50%.

### 2.3. Study Instruments

After signing the consent form, students were tested for antibodies against *H. pylori* using serological tests from finger capillary blood. PRIMA^®^, a self-test for the detection of *H. pylori* antibodies in human whole blood samples, was used. This is an immunochromatographic test that detects *H. pylori* IgG antibodies in blood using special gold-conjugated monoclonal antibodies integrated in the reaction strip. According to the manufacturer, the specificity of this test is 84.2%, the sensitivity is 95.8%, and the accuracy is 87% [13]. All tests were performed according to the given manufacturer’s instructions.

Following the given specificity (Sp) and sensitivity (Se) and the observed prevalence of *H. pylori* infection in the study sample (P), we calculated the positive predictive value (PPV) of the serological test using the following formula:

PPV = (Se × P)/[(Se × P) + (1 − Sp) × (1 − P)].

PPV measures the probability that a person who receives a positive test result truly has the condition [14].

Additionally, after performing the *H. pylori* test, students completed a questionnaire. The questions, response options, and categorization of answers used in this study are presented in Supplementary Table S1.

The questionnaire included the 15-item Gastrointestinal Symptoms Rating Scale (GSRS). This scale was designed to assess the prevalence and intensity of gastrointestinal symptoms among students. It consisted of a seven-point Likert-type scale, where 0 indicated the absence of symptoms, and 6 indicated very troublesome symptoms. The total GSRS score was calculated as the sum of all 15 item scores. In addition, the sum was computed for specific symptom groups as determined in the analyses. Higher total or subgroup scores indicated more severe symptoms. The reliability and validity of the GSRS have been well documented [12,15]. The overall content validity of the questionnaire was ensured through careful review, and consensus was reached among the research team to confirm clarity, relevance, and alignment with the study objectives.

All study variables were collected based on self-reported information provided by the students, including gastrointestinal symptoms, oral health status, oral hygiene behaviors, and relevant background characteristics; no clinical examinations were performed.

### 2.4. Statistical Analysis

Descriptive statistics were calculated to estimate the frequency (n), percentage (%), mean, standard deviation (SD), range of the variables, and the 95% confidence interval (CI) for the mean. The normality of the variable distribution was tested using the Kolmogorov–Smirnov test. When normality was rejected, the Mann–Whitney test was applied to compare the distributions of the variables. The Bonferroni z-test was applied to compare the percentages of categorical variables. Spearman’s correlation coefficient (ρ) and the Chi-square (χ^2^) test were used to evaluate the bivariate associations between variables. Odds ratios (ORs) with 95% CI were calculated to evaluate the risk of *H. pylori* occurrence in multivariate analysis. A *p* value < 0.05 was considered statistically significant.

Data analysis also included an assessment of the psychometric properties of the GSRS. A set of these properties, as outlined by Boateng et al. (2018) [16], including Cronbach’s alpha for internal consistency reliability, was calculated. An alpha value greater than 0.8 was considered indicative of good reliability. In the present study, the GSRS had very good internal consistency, with an overall Cronbach’s Alpha of 0.860.

An Exploratory Factor Analysis (EFA) was performed on the set of GSRS items. The suitability of the data for this analysis was evaluated using the Kaiser–Meyer–Olkin (KMO) measure of sampling adequacy and Bartlett’s test of sphericity. A KMO value ≥ 0.5 and *p* < 0.001 indicated that the data were adequate for EFA. Due to the asymmetric distribution of items in the scale, a Principal Axis Factoring approach was applied for factor extraction, as this method is more robust to deviations from normality [17]. Initially, a single-factor solution was explored to rank the items by their contribution to the total variance of the scale. Subsequently, based on the eigenvalue criterion (>1), four factors were extracted. A Varimax rotation with Kaiser normalization was employed. This procedure enabled the calculation of factor loadings for each item in a meaningful way, as the extracted factors were uncorrelated [18]. Factor loadings below 0.4 were considered to indicate low item contribution to the validity of the instrument; therefore, further analyses were conducted without such items. All analyses were conducted using SPSS statistical software (version 21; IBM SPSS Inc., Chicago, IL, USA) [18].

## 3. Results

### 3.1. Study Population Characteristics

The study population comprised 202 dentistry students, comprising 30 (14.9%) males and 172 (85.1%) females; 97 (48%) and 105 (52%) students were enrolled in lower (I or II) and higher (IV or V) academic years, respectively (Table 1).

Students from higher academic years, compared to those from lower academic years, paid greater attention to oral hygiene. They were more likely to brush their teeth for a longer duration (3 min or more) (38.1% vs. 24.7%, *p* = 0.042), use an irrigator for cleaning (34.3% vs. 19.6%, *p* = 0.019), and use toothpaste containing fluoride (98.1% vs. 93.1%, *p* = 0.003). A smaller proportion of higher-year students had caries-damaged teeth (18.1% vs. 32.0%, *p* = 0.023), while a larger proportion had filled teeth (67.6% vs. 41.2%, *p* < 0.001). They also reported slightly fewer cases of bleeding gums (4.8% vs. 11.3%, *p* = 0.084).

Among dentistry students, those from higher academic years had significantly more experience in treating patients or assisting a dentist compared to students from lower academic years. Consequently, they more frequently used protective equipment such as a mask (100% vs. 70.1%, *p* < 0.001), gloves (100% vs. 70.1%, *p* < 0.001), a face shield (25.7% vs. 10.3%, *p* = 0.005), and protective glasses (32.4% vs. 15.4%, *p* = 0.005).

Table 1 also presents the characteristics of the investigated students according to other factors, none of which showed significant differences between students from the lower and higher academic years.

### 3.2. Seroprevalence of H. pylori and Associated Factors

In total, a seropositive *H. pylori* test in the study population was found in 25 (12.4%) dentistry students. Based on the observed prevalence and the manufacturer’s [13] reported sensitivity (95.8%) and specificity (84.2%) of the serological test, the estimated positive predictive value (PPV) was 46.2%.

The prevalence of seropositive *H. pylori* tests did not differ significantly across groups by academic year (*p* = 0.650). Table 2 displays the association between the prevalence of *H. pylori* and other characteristics of the investigated students.

Most of these characteristics were not significantly associated with a seropositive *H. pylori* test. Only two characteristics were found to be significantly (*p* < 0.05) associated with *H. pylori* occurrence: toothbrushing duration and family history of stomach ulcer.

Among the evaluated oral hygiene behaviors, only toothbrushing duration showed a statistically significant association with serological status (*p* = 0.020), whereas no significant differences were found for toothbrushing frequency or other behavioral variables. Brushing for 3 min or longer was more common among seropositive participants (52.0%) compared to seronegative participants (28.8%); in other words, students who brushed their teeth for at least 3 min were more likely to be seropositive than those who brushed for less time (20.3% vs. 8.7%, respectively; *p* = 0.020). The significance of this relationship was verified by adjusting data for students’ gender and study grade: OR = 2.76, 95% CI: 1.16–6.56, *p* = 0.022.

The second characteristic was a family history of stomach ulcer, which was reported significantly more often among seropositive students compared to seronegative students (36.0% vs. 18.1%, respectively; *p* = 0.037). The significance of this relationship was also verified by adjusting data for students’ gender and study grade: OR = 2.84, 95% CI: 1.12–7.16, *p* = 0.027.

In addition, it is worth noting several other participant characteristics, such as male gender, smoking habit, and self-reported presence of currently caries-damaged teeth and bleeding gums, which were associated with a higher likelihood of *H. pylori* seropositivity; however, these associations did not reach statistical significance due to the limited sample size and reduced statistical power.

Finally, based on the presented data, it is important to emphasize that *H. pylori* seropositivity among dental students is not associated with the level of clinical exposure during dental training and the use of protective tools.

### 3.3. Assessment of the Gastrointestinal Symptoms Rating Scale

Respondents rated the scale items from 0 to 6 points, except for S3 (Regurgitation), to which no respondent gave a score of 5 or 6. The highest levels of complaints were reported for S6 (Borborygmus) and S7 (Epigastric fullness), with mean scores of 1.50 and 1.42, respectively. The lowest levels of complaints were related to S3 (Regurgitation) and S5 (Nausea), with mean scores of 0.48 and 0.49, respectively.

The total GSRS score ranged from 0 to 63, with a mean of 12.73 (SD = 11.20) and a median of 9.50. The One-sample Kolmogorov–Smirnov test (*p* = 0.001) indicated that the null hypothesis of normality for the total score distribution should be rejected.

A more detailed analysis of the GSRS revealed a four-factor structure of the scale (Table 3). Each factor represented a distinct group of related gastrointestinal symptoms. Factor 1 reflected constipation-related symptoms (S10, S13, S15), Factor 2 represented indigestion or upper abdominal discomfort, including sensations such as rumbling, fullness, and flatulence (S6, S7, S4, S8, S9), Factor 3 captured diarrhea-related symptoms (S12, S11, S14), and Factor 4 focused on reflux-related complaints such as heartburn and regurgitation (S2, S3). The factor loadings ranged from 0.477 to 0.917, demonstrating satisfactory construct validity and clear item clustering. The four-factor structure explained 51% of the total variation in the GSRS item variation.

Both items S1 (Pain or discomfort) and S5 (Nausea) demonstrated low factor loadings across all extracted factors (<0.40), indicating that they did not align strongly with any of the four identified factors. This pattern suggests that these items represent more general or nonspecific gastrointestinal symptoms rather than belonging to a distinct symptom factor such as constipation, indigestion, diarrhea, or reflux. Although their contribution to the factorial structure of the GSRS was limited, both items were retained in the scale due to their clinical relevance and their role in capturing the broader spectrum of gastrointestinal complaints.

### 3.4. Trend of the Dyspeptic Symptoms by Academic Year

Table 4 presents the comparison of GSRS sum scores between dental students of lower (I and II) and higher (IV and V) academic years. The overall GSRS score was significantly higher among students in grades IV and V (Mean = 14.63 ± 11.83) compared to those in grades I and II (Mean = 10.67 ± 10.13; *p* = 0.011), indicating a greater burden of gastrointestinal symptoms in higher-grade students. Among the specific symptom factors, constipation-related symptoms (Mean = 2.59 ± 3.34 vs. 1.67 ± 2.61; *p* = 0.047) and indigestion-related symptoms (Mean = 7.41 ± 5.88 vs. 5.44 ± 4.61; *p* = 0.025) were also significantly higher among students in the higher grades. In contrast, differences in diarrhea-related (*p* = 0.188), reflux-related (*p* = 0.434), epigastric pain or discomfort (*p* = 0.219), and nausea (*p* = 0.355) scores were not statistically significant. These findings suggest that gastrointestinal discomfort, particularly constipation and indigestion, tends to increase with academic year.

### 3.5. Association Between the Gastrointestinal Symptoms and H. pylori Occurrence

Table 5 presents a comparison of the GSRS sum scores between students with seronegative and seropositive *H. pylori* test results. Across all symptom domains, including constipation-related, indigestion-related, diarrhea-related, and reflux-related symptoms, as well as individual items such as epigastric pain and nausea, no statistically significant differences were observed between the two groups (all *p* > 0.05). The mean total GSRS score was 12.67 ± 11.48 for the seronegative group and 13.12 ± 9.41 for the seropositive group, indicating similar overall gastrointestinal symptom burden. These findings suggest that *H. pylori* seropositivity among the students in this study was not associated with increased severity or frequency of gastrointestinal symptoms.

## 4. Discussion

### 4.1. Seroprevalence of H. pylori Infection in Dental Students

In the current study, the seroprevalence of *H. pylori* among dental students in Lithuania was evaluated, investigating how it was associated with oral health indicators, gastrointestinal symptoms, and clinical exposure during dental training. The results showed that there was no significant difference between students in lower and higher academic years, and the overall seroprevalence of *H. pylori* was 12.4%. This prevalence is relatively low in comparison to the global prevalence. This prevalence is also consistent with recent research confirming that young adults and students have lower rates of *H. pylori* infection than the general adult population [1,19,20]. According to data from large epidemiological studies, the prevalence of *H. pylori* is significantly age-dependent and tends to rise with age, reflecting both cohort effects and cumulative lifetime exposure [2,21].

In recent decades, *H. pylori* prevalence in many Western countries has decreased, particularly among younger cohorts, which has been associated with improvements in living conditions, hygiene, and widespread antibiotic use [22]. However, the prevalence remains elevated in developing countries [22]. The relatively low seroprevalence in the present study’s cohort may therefore reflect broader epidemiological trends rather than characteristics specific only to dental students. For example, a study conducted with medical and nursing students studying in the Lithuanian University of Health Sciences has also shown a significantly lower percentage (14.2%) of prevalence compared to the overall Lithuanian population [12].

At the same time, a recent Lithuanian population-based study demonstrated significantly higher seroprevalence among adults, indicating that *H. pylori* infection remains common in individuals of older age within the same geographical setting [7]. Similarly, neighboring countries of Lithuania, such as Poland and Latvia, report similar or even higher prevalence of *H. pylori* infection, reaching up to 84% and 55%, respectively [23,24]. Older age as an important factor for *H. pylori* prevalence has also been demonstrated in a study, where people in the age group of 20–29 years old and a group of >50-year-olds had a prevalence of >23% and >50%, respectively [25]. In conclusion, the results of this study provide new knowledge on students from Eastern Europe and support the global trend of lower *H. pylori* prevalence in modern young adult populations.

### 4.2. Clinical Exposure and Occupational Risk

The idea that students in higher academic years would show a higher seroprevalence of *H. pylori* due to increased clinical exposure was one of the study’s main hypotheses. The present study findings contradicted this theory since there was no association found between serological status and the number of patients treated, the academic year, dental assistance, or personal protective equipment use. However, these findings should be interpreted cautiously. Because the study was conducted at a single academic center, the results may not be generalizable to other dental student populations or training environments. Some researchers have hypothesized that because dental professionals frequently come into contact with saliva, plaque, and aerosols, they should be regarded as a possible occupational risk group for *H. pylori* [20,26]. According to the scientific literature, as *H. pylori* can colonize the oral cavity, it can be disseminated during dental procedures with the use of aerosol [27,28]. Evidence supporting this theory and the occupational risk for dental professionals, however, is still inconsistent across the literature and is heavily impacted by national infection-control practices and baseline population prevalence. Some studies on practicing dentists report higher detection of oral *H. pylori* compared to control groups, which suggests that exposure is an important factor for long-term clinical professionals, but not so much for students, who have had a much shorter exposure duration [26]. To summarize, current research emphasizes that *H. pylori* is most often acquired via family members or intimate partners, through saliva, kissing, and sharing utensils, and does not typically categorize dental training as one of the main risk factors for *H. pylori* infection [29]; however, further research is necessary to further verify this.

### 4.3. Oral Hygiene and Oral Health Factors

One of the objectives in this study was to investigate dental hygiene practices as possible indicators of *H. pylori* seropositivity. Interestingly, there was no difference observed between *H. pylori*-positive and -negative students in most self-reported oral hygiene habits (frequency of brushing, use of floss or irrigator, etc.). An unexpected association was observed between *H. pylori* seropositivity and toothbrushing duration ≥3 min: students who brushed their teeth for at least 3 min were more likely to be seropositive than those who brushed for less time (20.3% vs. 8.7%, *p* = 0.020). This finding opposes the hypothesis that increased oral hygiene would reduce the risk of infection. Given the cross-sectional design of the study, the association does not show causality, but rather the reverse. Therefore, this result should be considered exploratory and requires confirmation in future studies with larger samples and a more detailed assessment of oral hygiene behaviors. Recent reviews also emphasize that the “oral reservoir” hypothesis is still up for discussion, with wide variation in reported oral *H. pylori* detection depending on sampling site, laboratory methods, and the definition of gastric infection status [3].

Regarding associations between oral health and *H. pylori*, a 2023 meta-analysis showed that there is a clear connection between periodontal disease and *H. pylori* infection [10]. Because periodontal diseases and *H. pylori* infection share several risk factors, such as socioeconomic status, poorer general health, smoking, alcohol use, and poorly controlled diabetes, having one condition may contribute to the development or worsening of the other [30,31]. Research also suggests that *H. pylori* may be associated with an increased risk of several oral conditions, including periodontal diseases, recurrent aphthous ulcers (canker sores), oral squamous cell carcinoma, tongue irritation, and halitosis [32,33]. In this context, one isolated association of *H. pylori* seropositivity with brushing duration can perhaps be explained by the fact that students possibly brush longer due to greater oral health concerns or other symptoms that they experience. Future studies that would include clinical oral examinations and standardized oral *H. pylori* testing (alongside active gastric infection testing) would be needed to clarify whether oral health significantly contributes to infection persistence or reinfection in similar populations.

### 4.4. Family History and Transmission Patterns

One of the other important findings was the association between *H. pylori* seropositivity and family history of stomach ulcers. Particularly, a family history of stomach ulcers was reported significantly more often among seropositive students compared to seronegative students (36.0% vs. 18.1%, *p* = 0.037). This finding may reflect current research suggesting that close contact intrafamilial transmission is a significant factor in *H. pylori* epidemiology, and that younger populations should be tested and treated to prevent transmission to their children [29,34]. Given the variety of different causes of ulcer disease, a family history of ulcers may indicate either a shared household exposure to *H. pylori* or a shared risk of non-infectious ulcer risk factors (e.g., similar use of NSAIDs in the family). In the present study, family history has shown a clearer association with seropositivity than variables related to exposure during dental procedures. Due to the cross-sectional study design and limited sample size, the results do not allow definitive conclusions regarding the relative importance of occupational versus household transmission pathways. Nevertheless, the observed association might suggest that household transmission routes could possibly be one of the most important even in groups involved in healthcare training.

### 4.5. Gastrointestinal Symptoms and Academic Year

A significant difference was found in the symptom burden between students in lower and higher years using the Gastrointestinal Symptoms Rating Scale (GSRS). Senior students had significantly higher overall GI symptom scores (mean GSRS 14.63 in 4th/5th year) than juniors (mean 10.67 in 1st/2nd year; *p* = 0.011). Specifically, seniors reported symptoms of constipation and indigestion more frequently than juniors (both groups, *p* < 0.05). Reflux, diarrhea, and nausea scores did not differ significantly, but the trend across different domains suggests that upper-year students have higher levels of gastrointestinal discomfort. This finding could reflect that gastrointestinal symptom burden may increase as students progress through training, which is consistent with studies linking student GI complaints to stress, irregular eating, and lifestyle changes [35]. Recent studies on student populations show that mental health factors are linked to functional gastrointestinal disorders (disorders of gut–brain interactions), suggesting that GI symptoms may be caused by stress and other psychosocial factors even without the presence of infection [36]. Because factors such as academic workload, psychological stress, dietary habits, and sleep patterns were not directly measured in this study, their potential contribution to gastrointestinal symptoms cannot be determined. Nevertheless, previous studies suggest that academic-year-related differences might be linked to study-related lifestyle changes and psychosocial stress rather than to an infection-driven cause.

### 4.6. Association Between H. pylori Seropositivity and Gastrointestinal Symptoms

In the present study, it was analyzed whether *H. pylori* seropositivity correlated with the severity of gastrointestinal symptoms in the student cohort. Interestingly, no significant differences in GSRS symptom scores between *H. pylori*-seropositive and -seronegative individuals were found. Infected students did not report more dyspeptic symptoms than non-infected students. Their total GSRS scores were almost the same (mean 13.12 vs. 12.67, *p* = 0.623). This applied to specific symptom subscales as well: constipation, indigestion, reflux, abdominal pain, and nausea scores were all statistically equivalent between the two groups. In other words, *H. pylori* was not associated with any increase in gastrointestinal symptom intensity in this young adult cohort.

These findings are consistent with the Maastricht VI/Florence consensus report, which states that patients with diagnosed *H. pylori* infection are frequently asymptomatic despite the presence of chronic gastritis caused by the infection [34]. A large cross-sectional study conducted in Lithuania likewise reported no statistically significant association between seroprevalence and gastrointestinal/dyspeptic complaints [7]. Although evidence does suggest that *H. pylori* is not the primary cause of dyspeptic symptoms, a 2019 meta-analysis found that eradication therapy produced a small but statistically significant increase in symptom relief compared to placebo (risk ratio (RR) = 1.18; 95% CI 1.07–1.30). In addition, with a number needed to treat (NNT) of 15, the benefit of *H. pylori* eradication on dyspeptic symptoms appears to be small [37]. However, when the *H. pylori* infection leads to peptic ulcer disease, symptoms typically become more pronounced, often needing further diagnostic investigation and eradication treatment [34]. These findings suggest that routine testing for *H. pylori* infection based solely on non-specific symptoms in otherwise healthy young individuals is not recommended. Instead, testing should be considered in the presence of persistent dyspepsia or relevant risk factors, with treatment initiated when infection is confirmed.

### 4.7. Study Limitations and Future Practical Implications

The first limitation, perhaps the most important, is related to *H. pylori* infection detection using only serological testing, which has certain disadvantages such as reflection of a past exposure or false-positive results due to individual variations in immune response [38]. The interpretation of the serological findings should also take into account the diagnostic characteristics of the assay and the relatively low prevalence of infection in the study population. Using the reported sensitivity (95.8%) and specificity (84.2%), together with the observed prevalence of *H. pylori* infection (12.4%), the estimated positive predictive value (PPV) was approximately 46%. In low-prevalence settings, even tests with high sensitivity may yield a substantial proportion of false-positive results because the number of true positive cases is relatively small compared to the number of non-infected individuals [14]. Therefore, the observed seroprevalence should be interpreted cautiously, as some positive serological results may reflect false positives or past exposure rather than current active infection. For future research, urea breath tests, stool antigen tests, and direct detection of *H. pylori* using polymerase chain reaction could be used as complementary methods. Combining serological testing with one or more of these approaches could lead to improved accuracy and provide a clearer picture of infection.

Secondly, the current research findings should be interpreted with caution because only approximately 200 students were included in the final analysis. The limited number of subjects reduced the statistical power to detect smaller or more subtle associations. Future studies with larger sample sizes would be necessary to confirm the presented findings.

The third limitation is related to the generalizability of the study findings, as they are based solely on dentistry students from a single university. The absence of a control group from other study programs limits the ability to determine whether the observed *H. pylori* seroprevalence is specific to dental students or reflects the general prevalence among university students.

Furthermore, it is worth mentioning that a cross-sectional study design is able to provide a snapshot of serological status among the studied group. However, because exposure variables and serological status are assessed simultaneously, the design does not allow for conclusions regarding causality. Given that *H. pylori* infection may persist asymptomatically for years, establishing causal relationships would require longitudinal follow-up or alternative study designs. However, despite this limitation, cross-sectional studies remain a practical initial step in exploring health trends and generating ideas for future research.

Another limitation relates to the measurement of occupational exposure during dental training. In the present study, clinical exposure was assessed using indirect indicators such as the number of patients treated, experience assisting in dental procedures, and the reported use of personal protective equipment. Although these variables provide a general indication of students’ clinical involvement, they may not fully capture the true level of exposure to saliva or aerosols. In particular, the type, frequency, and duration of specific dental procedures, which may influence exposure risk, were not assessed. Future studies incorporating more detailed measures of clinical activity and procedure-related exposure would help to better evaluate the potential occupational risk of *H. pylori* transmission in dental settings.

The practical application of the present findings is primarily related to dentistry students’ health status and their clinical evaluation. The number of *H. pylori*-seropositive results in this cohort was low, showing no relation with clinical dental training or gastrointestinal symptoms. The results do not support routine *H. pylori* testing or any other occupational precautions for dental students beyond standard infection-control measures that already exist in the clinic. The only factor that was associated with seropositivity was a reported family history of stomach ulcers, suggesting that individual clinical history would help in the early identification of individuals with a higher risk of *H. pylori* infection. In addition, senior students in this cohort showed a higher prevalence of gastrointestinal symptoms, independent of *H. pylori* serological status. This pattern, in which factors related to academic workload and student lifestyle are more important for the gastrointestinal symptoms than the infection itself, further supports the association between stress and gastrointestinal symptoms [35]. Together, these findings indicate that, in comparable dental student populations, prioritizing family history when evaluating for *H. pylori* may improve detection and clinical relevance more effectively than routine screening alone. They also highlight the importance of paying closer attention to students’ psychological wellbeing and related gastrointestinal symptoms, as supporting these factors is likely to contribute meaningfully to the overall health and wellbeing of dental students.

## 5. Conclusions

This study provides new evidence on the seroprevalence of *Helicobacter pylori* among Lithuanian dental students across academic years and its associations with gastrointestinal symptoms and self-perceived oral health. Overall, *H. pylori* seropositivity was detected in 12.4% of students. Seropositivity was not associated with increased clinical exposure over the course of dental training, suggesting that occupational exposure may have a limited role in infection risk, possibly due to the relatively short duration of clinical practice and limited cumulative exposure to patients at this stage of training. However, due to the single-center design and the relatively limited sample size, these results should be interpreted cautiously and require confirmation in larger multicenter studies. Among the examined variables, only one oral health behavior—brushing teeth for longer than three minutes—and a family history of stomach ulcers were associated with *H. pylori* seropositivity. No evidence of consistent association between seroprevalence of *H. pylori* and gastrointestinal symptoms or self-perceived oral health was observed. These findings highlight the need for continued investigation of *H. pylori* prevalence among dental students to improve understanding of its epidemiology and to inform more effective strategies for infection management.

## Figures and Tables

**Table 1:** Characteristics of investigated students by academic year.

Characteristic	Total Sample		Academic Year I and II		Academic Year IV and V		*p* Value ^1^
	n	(%)	n	(%)	n	(%)	
All respondents ^2^	202	(100.0)	97	(48.0)	105	(52.0)	0.622
Gender:							
Female	172	(85.1)	79	(81.4)	93	(88.6)	0.155
Male	30	(14.9)	18	(18.6)	12	(11.4)	
Living area during childhood:							
Urban	144	(71.3)	69	(71.1)	75	(71.4)	0.963
Rural	58	(28.7)	28	(28.9)	30	(28.6)	
Smoking habit:							
No	155	(76.7)	70	(72.2)	85	(81.0)	0.140
Yes	47	(23.3)	27	(27.8)	20	(19.0)	
Use of alcohol:							
Almost never	60	(29.3)	31	(32.0)	29	(27.6)	0.624
Occasionally	131	(64.9)	62	(63.9)	69	(65.7)	
Regularly	11	(5.4)	4	(4.1)	7	(6.7)	
Milk tolerance:							
Yes	152	(75.2	78	(78.4)	76	(72.4)	0.326
No	50	(24.8)	21	(21.8)	29	(27.5)	
Toothbrushing regularity:							
1–2 times/day	192	(90.1)	90	(92.8)	92	(87.6)	0.220
3 or more times/day	20	(9.9)	7	(7.2)	13	(12.4)	
Toothbrushing duration:							
Up to 3 min	138	(68.3)	73	(75.3)	65	(61.9)	**0.042**
3 min or longer	64	(31.7)	24	(24.7)	40	(38.1)	
Using an irrigator:							
No	147	(72.8)	78	(80.4)	69	(65.7)	**0.019**
Yes	55	(27.2)	19	(19.6)	36	(34.3)	
Using toothpaste with fluoride:							
No	14	(6.9)	12	(12.4)	2	(1.9)	**0.003**
Yes	188	(93.1)	85	(87.6)	103	(98.1)	
Currently caries-damaged teeth:							
No	152	(75.2)	66	(68.0)	86	(81.9)	**0.023**
Yes	50	(24.8)	31	(32.0)	19	(18.1)	
Number of filled teeth:							
Up to 4	91	(45.0)	57	(58.8)	34	(32.4)	**<0.001**
5 or more	111	(55.0)	40	(41.2)	71	(67.6)	
Bleeding gums:							
No	186	(92.1)	86	(88.7)	100	(95.2)	0.084
Yes	16	(7.9)	11	(11.3)	5	(4.8)	
Number of patients treated:							
Up to 10	96	(47.5)	85	(87.6)	11	(10.5)	**<0.001**
11 or more	106	(52.5)	12	(12.4)	94	(89.5)	
Dental assistance:							
Never	69	(34.2)	55	(56.7)	14	(13.3)	**<0.001**
Several times	133	(65.8)	42	(43.3)	91	(86.7)	
Using a protective mask:							
No	29	(14.4)	29	(29.9)	0		**<0.001**
Yes	173	(85.6)	68	(70.1)	105	(100.0)	
Using protective gloves:							
No	29	(14.4)	29	(29.9)	0		**<0.001**
Yes	173	(85.6)	68	(70.1)	105	(100.0)	
Using a protective shield:							
No	165	(81.7)	87	(89.7)	78	(74.3)	**0.005**
Yes	37	(18.3)	10	(10.3)	27	(25.7)	
Using safety glasses:							
No	153	(75.7)	82	(84.5)	71	(67.6)	**0.005**
Yes	49	(24.3)	15	(15.5)	34	(32.4)	
Family history of stomach ulcer:							
No	161	(79.7)	81	(83.5)	80	(76.2)	0.197
Yes	41	(20.3)	16	(16.5)	25	(23.8)	
Family history of gastric cancer:							
No	177	(87.6)	87	(89.7)	90	(85.7)	0.391
Yes	25	(12.4)	10	(10.3)	15	(14.3)	
Test for *H. pylori*:							
Seronegative	177	(87.6)	83	(86.5)	93	(88.6)	0.650
Seropositive	25	(12.4)	13	(13.5)	12	(12.4)	

Notes: ^1^, Chi-squared test; ^2^, One-sample test. Bold values indicate statistically significant results (*p* < 0.05).

**Table 2:** Association between prevalence of *H. pylori* and characteristics of investigated students.

Characteristic	Seronegative Test (n = 177)		Seropositive Test (n = 25)		*p* Value ^1^
	n	(%)	n	(%)	
Gender:					
Female	153	(89.0)	19	(76.0)	0.169
Male	24	(13.6)	6	(24.0)	
Living area during childhood:					
Urban	128	(72.3)	16	(64.0)	0.390
Rural	49	(27.7)	9	(36.0)	
Smoking habit:					
Yes	39	(22.0)	8	(32.0)	0.270
Use of alcohol:					
Almost never	54	(30.7)	6	(24.0)	0.724
Occasionally	113	(63.6)	18	(72.0)	
Regularly	10	(5.7)	1	(4.0)	
Milk tolerance:					
No	42	(23.7)	8	(32.0)	0.370
Toothbrushing regularity:					
1–2 times/day	159	(90.3)	22	(88.0)	0.704
3 or more times/day	17	(9.6)	3	(12.0)	
Toothbrushing duration:					
Up to 3 min	126	(71.2)	12	(48.0)	**0.020**
3 min or longer	51	(28.8)	13	(52.0)	
Using an irrigator:					
Yes	48	(27.1)	7	(28.0)	0.926
Using toothpaste with fluoride:					
No	11	(6.2)	3	(12.0)	0.286
Decay in primary teeth:					
Yes	24	(13.6)	3	(12.0)	0.830
Currently caries-damaged teeth:					
Yes	42	(23.7)	8	(32.0)	0.370
Number of filled teeth:					
5 or more	95	(53.7)	16	(64.0)	0.331
Bleeding gums:					
Yes	12	(6.8)	4	(16.0)	0.110
Number of patients treated:					
11 or more	93	(52.5)	13	(52.0)	0.959
Dental assistance:					
Never	59	(33.3)	10	(40.0)	0.511
Using a protective mask:					
No	25	(14.1)	4	(16.0)	0.802
Using protective gloves:					
No	25	(14.1)	4	(16.0)	0.802
Using a protective shield:					
No	144	(81.4)	21	(84.0)	0.749
Using safety glasses:					
No	136	(76.8)	17	(68.0)	0.335
Family history of stomach ulcer:					
Yes	32	(18.1)	9	(36.0)	**0.037**
Family history of gastric cancer:					
Yes	22	(12.4)	3	(12.0)	0.951
Personal stomach ulcer:					
Yes	6	(3.4)	0	(0)	0.349
Previously tested for *H. pylori*:					
Was negative	29	(16.4)	4	(16.0)	0.565
Was positive	11	(6.3)	3	(12.0)	
Not tested	137	(77.3)	18	(72.0)	
Took medication for *H. pylori*:					
Yes	9	(5.1)	2	(8.0)	0.504

Note: ^1^ Chi-squared test. Bold values indicate statistically significant results (*p* < 0.05).

**Table 3:** Rotated factor matrix of the Gastrointestinal Symptoms Rating Scale (N = 202).

	Factor			
	1	2	3	4
S10 ^1^ Constipation	0.917	0.136	0.071	0.078
S13 ^1^ Troubled by passing hard stools	0.682	0.142	0.087	0.175
S15 ^1^ Incomplete bowel emptying	0.565	0.191	0.135	0.072
S6 ^2^ Borborygmus	0.161	0.656	0.131	0.076
S7 ^2^ Epigastric fullness	0.302	0.615	0.077	0.151
S4 ^2^ Hunger-like pain	−0.029	0.588	0.224	0.208
S8 ^2^ Belching	0.256	0.487	0.059	0.338
S9 ^2^ Flatulence	0.370	0.477	0.356	0.181
S5 Nausea	0.189	0.291	0.158	0.199
S12 ^3^ Troubled by passing loose stools	0.160	0.048	0.815	0.167
S11 ^3^ Diarrhea	0.125	0.168	0.754	0.067
S14 ^3^ Sudden strong urge to defecate	0.033	0.207	0.583	0.099
S2 ^4^ Heartburn	0.071	0.196	0.168	0.903
S3 ^4^ Regurgitation	0.241	0.212	0.093	0.481
S1 Pain or discomfort	0.075	0.318	0.334	0.384

Notes: ^1–4^ Items assigned to Factors 1–4, respectively; the highest factor loadings are highlighted by shaded cells. Extraction Method: Principal Axis Factoring. Rotation Method: Varimax with Kaiser Normalization. KMO Measure of Sampling Adequacy was 0.809. Bartlett’s Test of Sphericity *p* < 0.001.

**Table 4:** Comparison of sum scores of the Gastrointestinal Symptoms Rating Scale by students’ academic year.

Groups of Symptoms	Academic Years	N	Mean	Std. Deviation	95% Confidence Interval for Mean		*p* Value ^1^
					Lower Bound	Upper Bound	
All items	I + II	97	10.67	10.13	8.63	12.71	**0.011**
	IV + V	105	14.63	11.83	12.34	16.92	
	Total	202	12.73	11.20	11.17	14.28	
Factor 1: Constipation-related symptoms (S10 + S13 + S15)	I + II	97	1.67	2.61	1.15	2.20	**0.047**
	IV + V	105	2.59	3.34	1.94	3.24	
	Total	202	2.15	3.04	1.73	2.57	
Factor 2: Indigestion-related symptoms (S6 + S7 + S4 + S8 + S9)	I + II	97	5.44	4.61	4.52	6.37	**0.025**
	IV + V	105	7.41	5.88	6.27	8.55	
	Total	202	6.47	5.39	5.72	7.21	
Factor 3: Diarrhea-related symptoms (S12 + S11 + S14)	I + II	97	1.39	2.62	0.86	1.92	0.188
	IV + V	105	1.94	3.03	1.36	2.53	
	Total	202	1.68	2.84	1.28	2.07	
Factor 4: Reflux-related symptoms (S2 + S3)	I + II	97	1.12	1.84	0.75	1.50	0.434
	IV + V	105	1.38	2.16	0.96	1.80	
	Total	202	1.26	2.01	0.98	1.54	
Pain or discomfort (S1)	I + II	97	0.61	1.24	0.36	0.67	0.219
	IV + V	105	0.77	1.30	0.52	1.02	
	Total	202	0.70	1.27	0.52	0.87	
Nausea (S5)	I + II	97	0.43	1.15	0.20	0.67	0.355
	IV + V	105	0.53	1.19	0.30	0.76	
	Total	202	0.49	1.17	0.32	0.65	

Note: ^1^ Mann–Whitney U test. Bold values indicate statistically significant results (*p* < 0.05).

**Table 5:** Comparison of sum scores of Gastrointestinal Symptoms Rating Scale between groups of students with seronegative and seropositive *H. pylori* test.

Groups of Symptoms	Test Result	N	Mean	Std. Deviation	95% Confidence Interval for Mean		*p* Value ^1^
					Lower Bound	Upper Bound	
All items	Seronegative	177	12.67	11.48	10.97	14.39	0.623
	Seropositive	25	13.12	9.41	9.23	17.01	
	Total	202	12.73	11.23	11.17	14.30	
Factor 1: Constipation-related symptoms (S10 + S13 + S15)	Seronegative	177	2.19	3.06	1.74	2.65	0.481
	Seropositive	25	1.92	2.96	0.70	3.14	
	Total	202	2.16	3.04	1.74	2.58	
Factor 2: Indigestion-related symptoms (S6 + S7 + S4 + S8 + S9)	Seronegative	177	6.32	5.40	5.51	7.12	0.273
	Seropositive	25	7.44	5.36	5.23	9.65	
	Total	202	5.46	5.40	5.71	7.21	
Factor 3: Diarrhea-related symptoms (S10 + S11 + S14)	Seronegative	177	1.68	2.88	1.25	2.10	0.918
	Seropositive	25	1.64	2.71	0.52	2.76	
	Total	202	1.67	2.85	1.28	2.07	
Factor 4: Reflux-related symptoms (S2 + S3)	Seronegative	177	1.27	2.00	0.97	1.56	0.675
	Seropositive	25	1.24	2.17	0.35	2.13	
	Total	202	1.26	2.02	0.98	1.54	
Pain or discomfort (S1)	Seronegative	177	0.70	1.26	0.52	0.89	0.541
	Seropositive	25	0.64	1.38	0.07	1.21	
	Total	202	0.70	1.27	0.52	0.87	
Nausea (S5)	Seronegative	177	0.52	1.22	0.34	0.70	0.297
	Seropositive	25	0.24	.72	0.00	0.54	
	Total	202	0.49	1.17	0.32	0.65	

Note: ^1^ Mann–Whitney U test.

## Data Availability

The data presented in this study are available from the corresponding author on request.

## Supplementary Materials

The following supporting information can be downloaded at https://www.mdpi.com/article/10.3390/diagnostics16071049/s1, Table S1: Variables of the study and their respective categorization.
