# Supplementary material for: Helicobacter pylori Seroprevalence and Its Association with Gastrointestinal Symptoms and Self-Perceived Oral Health Among Lithuanian Dental Students
Source: Diagnostics (Basel). 2026 Mar 31;16(7):1049. doi: 10.3390/diagnostics16071049 (PMC13072910; doi:10.3390/diagnostics16071049)
Supplement: Supplementary file 1 [file diagnostics-16-01049-s001.zip › diagnostics-4191370-supplementary.pdf]

Table S1. Variables of the study and their respective categorization.

Variable	Question	Response options and their categorization
Gender	What is your gender?	1=male; 2=female
Academic year	What course are you studying?	1=I / II; 2=IV / V
Living area during childhood	Where were you born and raised until you were 16?	1=urban area (Vilnius, Kaunas, Klaipėda, Šiauliai or Panevėžys / other town)<br>2=rural area (a small town / country)
Smoking habit	Do you smoke?	0=no; 1=yes
Using of alcohol	How often do you drink alcohol?	0=no (I don't use / 1–2 times a year)<br>1=yes (1–2 times a month / 1–2 times a week / more often)
Milk tolerance	Can you drink milk (unfermented) or consume milk products?	1=yes; 2=no (I can’t because it causes symptoms / I consume it in small amounts because it causes symptoms / I don’t like it)
Toothbrushing regularity	How many times a day do you brush your teeth?	1=1 time or 2 times<br>2=3 times or more
Toothbrushing duration	How long do you brush your teeth?	1= up to 3 minutes (up to 1 minute / 2–3 minutes); 2= 3 minutes or longer
Using an irrigator	Do you use an irrigator for oral care?	1=no; 2=yes
Using toothpaste with fluoride	Do you use toothpaste with fluoride?	1=no; 2=yes
Decay in primary teeth	Did you have severely decayed primary teeth in your primary bite with caries complications (pulpitis, periodontitis, only the roots remaining)?	1=no; 2=yes
Currently caries-damaged teeth	Do you have any caries-damaged teeth at the moment?	1=no; 2=yes (1–2 teeth / 3–4 teeth / more than 4 teeth)
Number of filled teeth	How many teeth do you currently have filled?	1=up to 4 (none / 1–2 teeth / 3–4 teeth)<br>2=5 or more
Bleeding gums	Do you currently have bleeding gums?	1=no; 2=yes
Number of patients treated	How many patients have you treated clinically?	1=up to 10 patients (none / up to 10); 2=11 or more patients (10–20 / more than 20)
Dental assistance	Do you additionally assist in a dental office outside of the university before or during your studies?	1=no; 2=yes
Using a protective mask	Do you use a protective mask when working with patients?	1=no; 2=yes
Using protective gloves	Do you use protective gloves when working with patients?	1=no; 2=yes
Using a protective shield	Do you use a protective shield when working with patients?	1=no; 2=yes
Using protective glasses	Do you use protective glasses when working with patients?	1=no; 2=yes
Family history of stomach ulcer	Do your relatives have (or have had) a stomach or duodenal ulcer?	1=no; 2=yes
Family history of gastric cancer	Do your relatives have (or have had) a gastric cancer?	1=no; 2=yes

Variable	Question	Response options and their categorization
Personal stomach ulcer	Do you have (have you had) a stomach or duodenal ulcer?	1=no; 2=yes
Other illnesses	Do you have any other illnesses?	1=no; 2=yes
Previously tested for *H. pylori*	Have you ever been diagnosed with *H. pylori*, if you have been tested for it?	1=was negative; 2=was positive<br>3=not tested
Took medication for *H. pylori*	Have you taken medications to eradicate *H. pylori*?	1=no; 2=yes
**Gastrointestinal Symptoms Rating Scale (GSRS)**		
S1 Pain or discomfort	During the past week, have you been bothered by **pain or discomfort** in the upper part of your abdomen or in the pit below your breastbone**?**	0=no symptoms<br>1=very mild symptoms<br>2=mild symptoms<br>3=moderate symptoms<br>4=moderately severe symptoms<br>5=severe symptoms<br>6=very severe symptoms
S2 Heartburn	…, have you been bothered by **heartburn** (an unpleasant burning or stinging sensation behind the breastbone)?	
S3 Regurgitation	…, have you been bothered by **acid regurgitation** (small amounts of acidic or bitter-tasting material coming up from the stomach into the throat or mouth)?	
S4 Hunger-like pain	…, have you been bothered by **hunger pains** in the upper part of your abdomen (a feeling of emptiness in the stomach associated with the need to eat frequently)?	
S5 Nausea	…, have you been bothered by **nausea** (an unpleasant sensation that may lead to hiccups or vomiting)?	
S6 Borborygmus	…, have you been bothered by **rumbling or gurgling sensations** (vibration or sounds) in your stomach?	
S7 Epigastric fullness	…, have you felt a **feeling of fullness in your stomach**? (This refers to a sensation of air in the stomach or abdominal bloating.)	
S8 Belching	…, have you been bothered by **belching**? (The release of air from the stomach through the mouth, associated with a sensation of stomach bloating.)	
S9 Flatulence	…, have you been bothered by the need to pass gas (**flatulence**)?	
S10 Constipation	…, have you been bothered by **constipation** (infrequent bowel movements)?	
S11 Diarrhea	…, have you been bothered by **diarrhea** (frequent bowel movements)?	
S12 Passing loose stools	…, have you been bothered by **passing loose stools**? (If loose stools alternate with hard ones, please indicate to what extent passing loose stools bothers you)	
S13 Passing hard stools	…, have you been bothered by **passing hard stools**? (If hard stools alternate with loose ones, please indicate to what extent passing hard stools bothers you)	
S14 Sudden strong urge to defecate	…, have you been bothered by a **sudden, strong urge to defecate**? (This urge is accompanied by a feeling that you can no longer control yourself)	
S15 Incomplete bowel emptying	…, have you felt a sensation **of incomplete bowel emptying**? (The feeling that, despite all efforts, you still have the urge to defecate after a bowel movement.)	
